# 1′-Phenyl-6′-thia­cyclo­heptane-1-spiro-2′-perhydro­pyrrolizine-3′-spiro-3′′-indoline-2,2′′-dione

**DOI:** 10.1107/S1600536807068705

**Published:** 2008-01-23

**Authors:** S. Sundaramoorthy, D. Gayathri, D. Velmurugan, M. Poornachandran, K. Ravikumar

**Affiliations:** a Centre of Advanced Study in Crystallography and Biophysics, University of Madras, Guindy Campus, Chennai 600 025, India; b Department of Organic Chemistry, University of Madras, Guindy Campus, Chennai 600 025, India; c Laboratory of X-ray Crystallography, Indian Institute of Chemical Technology, Hyderabad 500 007, India

## Abstract

The thia­zolidine ring and the pyrrolidine ring in the title compound, C_25_H_26_N_2_O_2_S, both adopt an envelope conformation. The seven-membered ring has a twist-chair conformation. The crystal packing is stabilized by inter­molecular N—H⋯O hydrogen bonds.

## Related literature

For related literature, see: Amal Raj *et al.* (2003 ▶); Cremer & Pople (1975 ▶); Kumar *et al*. (2006 ▶); Nardelli (1983 ▶); Si *et al.* (2005 ▶).
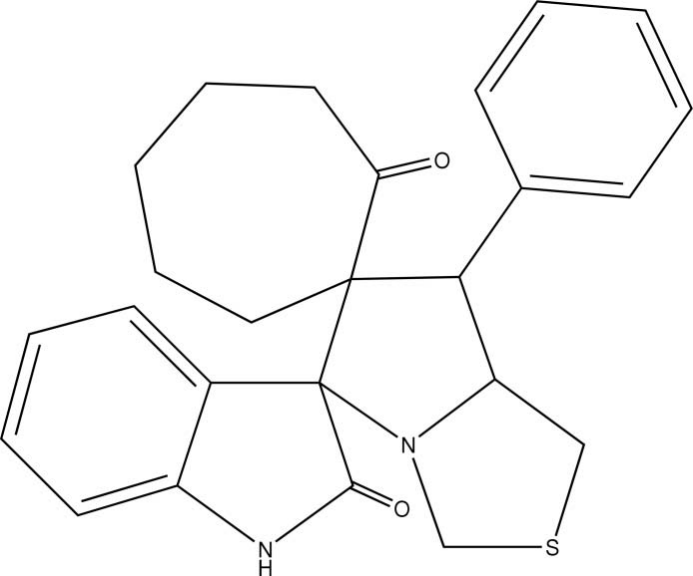


## Experimental

### 

#### Crystal data

C_25_H_26_N_2_O_2_S
*M* *_r_* = 418.54
Triclinic, 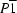

*a* = 8.9846 (10) Å
*b* = 10.3564 (11) Å
*c* = 12.8124 (14) Å
α = 80.147 (2)°
β = 71.012 (2)°
γ = 67.497 (2)°
*V* = 1040.0 (2) Å^3^
*Z* = 2
Mo *K*α radiation
μ = 0.18 mm^−1^
*T* = 293 (2) K
0.26 × 0.25 × 0.23 mm

#### Data collection

Bruker SMART CCD area-detector diffractometer
Absorption correction: none
11582 measured reflections
4697 independent reflections
3880 reflections with *I* > 2σ(*I*)
*R*_int_ = 0.023

#### Refinement

*R*[*F* ^2^ > 2σ(*F* ^2^)] = 0.062
*wR*(*F* ^2^) = 0.157
*S* = 1.08
4697 reflections
271 parameters
H-atom parameters constrained
Δρ_max_ = 0.36 e Å^−3^
Δρ_min_ = −0.20 e Å^−3^

### 

Data collection: *SMART* (Bruker, 2001 ▶); cell refinement: *SAINT* (Bruker, 2001 ▶); data reduction: *SAINT*; program(s) used to solve structure: *SHELXS97* (Sheldrick, 2008 ▶); program(s) used to refine structure: *SHELXL97* (Sheldrick, 2008 ▶); molecular graphics: *PLATON* (Spek, 2003 ▶); software used to prepare material for publication: *SHELXL97* and *PARST* (Nardelli, 1995 ▶).

## Supplementary Material

Crystal structure: contains datablocks I, global. DOI: 10.1107/S1600536807068705/bt2666sup1.cif

Structure factors: contains datablocks I. DOI: 10.1107/S1600536807068705/bt2666Isup2.hkl

Additional supplementary materials:  crystallographic information; 3D view; checkCIF report

## Figures and Tables

**Table 1:** Hydrogen-bond geometry (Å, °)

*D*—H⋯*A*	*D*—H	H⋯*A*	*D*⋯*A*	*D*—H⋯*A*
N2—H2⋯O1^i^	0.86	2.04	2.859 (2)	160

Symmetry code: (i) 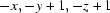.

**Fig. 1.**
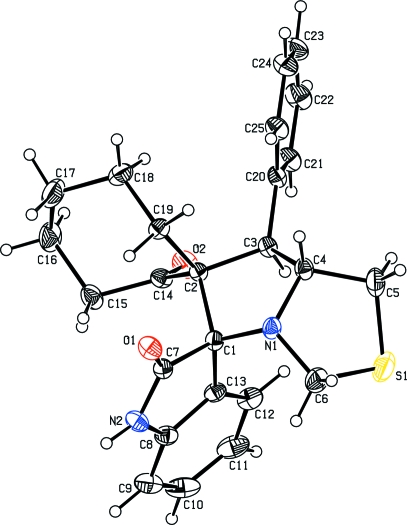
The molecular structure of the title compound, with atom labels and 30% probability displacement ellipsoids.

**Fig. 2.**
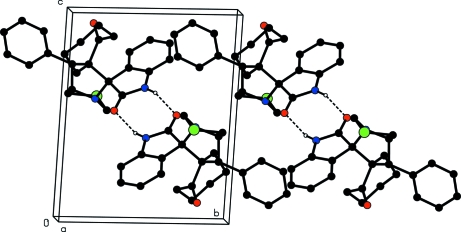
The packing of the title compound, viewed down the a axis, H atoms not involved in hydrogen bonding have been omitted.

## supplementary crystallographic information

### Comment

It has been reported that pyrrolidine derivatives reduce Coxsackievirus B3 replication through inhibition of the Ubiquitin-Proteasome pathway (Si *et al.*, 2005). They are found to have antimicrobial and antifungal activity against various pathogens except Bacillus subtilis (Amal Raj *et al.*, 2003). As the derivatives of pyrrolidine and oxindole are of pharmacological importance, we have undertaken the X-ray crystal structure determination of the title compound.

The bond lengths and bond angles of the title compound are comparable with a similar structure (Kumar *et al.*, 2006). The sum of the bond angles around N1 atom [341.8 (5)°] indicates *sp*^3^ hybridization. The dihedral angle between the phenyl ring and the six membered in the oxindole moiety is 40.5 (1)°.

The thiazolidine ring adopts an envelope conformation with S1 atom deviating by 0.764 (1) Å. The pyrrolidine ring (N1/C1—C4) adopts an envelope conformation with C2 atom deviating by -0.655 (2) Å. The puckering parameters (Cremer & Pople, 1975) and the smallest displacement asymmetry parameters (Nardelli, 1983) for the pyrrolidine ring, five membered ring (N2/C7/C1/C13/C8) in the oxindole moiety and the thiazolidine ring are q_2_ = 0.424 (2), 0.068 (2), 0.443 (2) Å, φ = 80.4 (3), 264.7 (18), 355.5 (3)° and Δ_s_(C_2_) = 7.7 (2), Δ_2_(N_2_) = 0.9 (3), Δ_s_(S_1_) = 5.9 (2).

The crystal packing is stabilized by intermolecular N—H···O hydrogen bonds generating a centrosymmetric dimeric ring motif [*R*_2_^2^(8)].

### Experimental

A mixture of isatin (0.147 g, 1 mmol), thiaproline, (0.135 g, 1 mmol) and benzylidenecycloheptanone (1 mmol) in methanol (20 ml) was refluxed until the disappearance of the starting materials. The reaction mixture was then concentrated *in vacuo* and extracted with water (50 ml) and dichloromethane (50 ml). The organic layer was washed with brine solution, dried with anhydrous sodium sulfate and concentrated *in vacuo*. The residue was purified by column chromatography with hexane-ethylacetate (8:2) mixture to get title compound. The pure compound was recrystallized from ethanol.

### Crystal data

**Table:**

C_25_H_26_N_2_O_2_S	*Z* = 2
*M**_r_* = 418.54	*F*_000_ = 444
Triclinic, *P*1	*D*_x_ = 1.337 Mg m^−^^3^
Hall symbol: -P 1	Mo *K*α radiation λ = 0.71073 Å
*a* = 8.9846 (10) Å	Cell parameters from 2358 reflections
*b* = 10.3564 (11) Å	θ = 1.7–25.0º
*c* = 12.8124 (14) Å	µ = 0.18 mm^−^^1^
α = 80.147 (2)º	*T* = 293 (2) K
β = 71.012 (2)º	Block, colorless
γ = 67.497 (2)º	0.26 × 0.25 × 0.23 mm
*V* = 1040.0 (2) Å^3^	

### Data collection

**Table:**

Bruker SMART CCD area-detector diffractometer	3880 reflections with *I* > 2σ(*I*)
Radiation source: fine-focus sealed tube	*R*_int_ = 0.023
Monochromator: graphite	θ_max_ = 28.0º
*T* = 293(2) K	θ_min_ = 1.7º
ω scans	*h* = −11→11
Absorption correction: none	*k* = −13→13
11582 measured reflections	*l* = −16→16
4697 independent reflections	

### Refinement

**Table:**

Refinement on *F*^2^	Secondary atom site location: difference Fourier map
Least-squares matrix: full	Hydrogen site location: inferred from neighbouring sites
*R*[*F*^2^ > 2σ(*F*^2^)] = 0.062	H-atom parameters constrained
*wR*(*F*^2^) = 0.157	*w* = 1/[σ^2^(*F*_o_^2^) + (0.0833*P*)^2^ + 0.2981*P*] where *P* = (*F*_o_^2^ + 2*F*_c_^2^)/3
*S* = 1.08	(Δ/σ)_max_ < 0.001
4697 reflections	Δρ_max_ = 0.36 e Å^−^^3^
271 parameters	Δρ_min_ = −0.20 e Å^−^^3^
Primary atom site location: structure-invariant direct methods	Extinction correction: none

### Special details

**Table:**

Geometry. All e.s.d.'s (except the e.s.d. in the dihedral angle between two l.s. planes) are estimated using the full covariance matrix. The cell e.s.d.'s are taken into account individually in the estimation of e.s.d.'s in distances, angles and torsion angles; correlations between e.s.d.'s in cell parameters are only used when they are defined by crystal symmetry. An approximate (isotropic) treatment of cell e.s.d.'s is used for estimating e.s.d.'s involving l.s. planes.
Refinement. Refinement of *F*^2^ against ALL reflections. The weighted *R*-factor *wR* and goodness of fit *S* are based on *F*^2^, conventional *R*-factors *R* are based on *F*, with *F* set to zero for negative *F*^2^. The threshold expression of *F*^2^ > σ(*F*^2^) is used only for calculating *R*-factors(gt) *etc*. and is not relevant to the choice of reflections for refinement. *R*-factors based on *F*^2^ are statistically about twice as large as those based on *F*, and *R*- factors based on ALL data will be even larger.

### Fractional atomic coordinates and isotropic or equivalent isotropic displacement parameters (Å^2^)

**Table:**

	*x*	*y*	*z*	*U*_iso_*/*U*_eq_	
C1	0.2327 (2)	0.71980 (19)	0.35784 (14)	0.0267 (4)	
C2	0.1643 (2)	0.84561 (18)	0.27522 (14)	0.0245 (4)	
C3	0.2629 (2)	0.93969 (19)	0.27834 (14)	0.0275 (4)	
H3	0.3807	0.8916	0.2386	0.033*	
C4	0.2543 (2)	0.9308 (2)	0.39991 (15)	0.0309 (4)	
H4	0.1503	1.0022	0.4386	0.037*	
C5	0.4043 (3)	0.9465 (3)	0.4219 (2)	0.0486 (6)	
H5A	0.3675	0.9961	0.4887	0.058*	
H5B	0.4568	0.9989	0.3607	0.058*	
C6	0.3716 (3)	0.7143 (3)	0.4993 (2)	0.0477 (6)	
H6A	0.4063	0.6146	0.4918	0.057*	
H6B	0.3256	0.7325	0.5773	0.057*	
C7	0.1116 (2)	0.63856 (19)	0.41978 (14)	0.0283 (4)	
C8	0.3423 (3)	0.4830 (2)	0.31052 (16)	0.0340 (4)	
C9	0.4440 (3)	0.3632 (2)	0.25409 (19)	0.0474 (6)	
H9	0.4118	0.2855	0.2648	0.057*	
C10	0.5958 (3)	0.3639 (3)	0.1810 (2)	0.0552 (7)	
H10	0.6665	0.2857	0.1404	0.066*	
C11	0.6440 (3)	0.4783 (3)	0.1674 (2)	0.0521 (6)	
H11	0.7482	0.4750	0.1193	0.063*	
C12	0.5404 (3)	0.5984 (2)	0.22390 (18)	0.0410 (5)	
H12	0.5741	0.6752	0.2141	0.049*	
C13	0.3852 (2)	0.6016 (2)	0.29553 (15)	0.0311 (4)	
C14	0.2143 (3)	0.79473 (19)	0.15917 (15)	0.0312 (4)	
C15	0.1212 (3)	0.7116 (2)	0.13959 (18)	0.0435 (5)	
H15A	0.0694	0.6711	0.2093	0.052*	
H15B	0.1990	0.6360	0.0920	0.052*	
C16	−0.0142 (4)	0.8082 (3)	0.0852 (2)	0.0584 (7)	
H16A	0.0370	0.8584	0.0216	0.070*	
H16B	−0.0576	0.7514	0.0588	0.070*	
C17	−0.1593 (3)	0.9132 (3)	0.1619 (2)	0.0600 (7)	
H17A	−0.2234	0.8631	0.2172	0.072*	
H17B	−0.2326	0.9763	0.1193	0.072*	
C18	−0.1110 (3)	1.0007 (2)	0.22123 (19)	0.0433 (5)	
H18A	−0.2113	1.0777	0.2538	0.052*	
H18B	−0.0339	1.0406	0.1669	0.052*	
C19	−0.0288 (2)	0.9197 (2)	0.31231 (15)	0.0307 (4)	
H19A	−0.0793	0.8497	0.3474	0.037*	
H19B	−0.0561	0.9844	0.3679	0.037*	
C20	0.2132 (2)	1.0876 (2)	0.22729 (15)	0.0300 (4)	
C21	0.3124 (3)	1.1172 (2)	0.12397 (17)	0.0393 (5)	
H21	0.4070	1.0459	0.0876	0.047*	
C22	0.2726 (3)	1.2504 (3)	0.07487 (19)	0.0503 (6)	
H22	0.3406	1.2681	0.0060	0.060*	
C23	0.1336 (4)	1.3570 (2)	0.1268 (2)	0.0512 (6)	
H23	0.1051	1.4461	0.0924	0.061*	
C24	0.0365 (3)	1.3313 (2)	0.2302 (2)	0.0477 (6)	
H24	−0.0565	1.4039	0.2665	0.057*	
C25	0.0765 (3)	1.1980 (2)	0.28063 (18)	0.0385 (5)	
H25	0.0110	1.1822	0.3511	0.046*	
N1	0.2471 (2)	0.79135 (17)	0.44146 (12)	0.0312 (4)	
N2	0.1822 (2)	0.50686 (17)	0.38528 (14)	0.0365 (4)	
H2	0.1347	0.4451	0.4066	0.044*	
O1	−0.02147 (18)	0.68469 (15)	0.49036 (11)	0.0377 (3)	
O2	0.3167 (2)	0.82744 (17)	0.08308 (11)	0.0449 (4)	
S1	0.55069 (8)	0.77332 (8)	0.43717 (6)	0.0609 (2)	

### Atomic displacement parameters (Å^2^)

**Table:**

	*U*^11^	*U*^22^	*U*^33^	*U*^12^	*U*^13^	*U*^23^
C1	0.0286 (9)	0.0307 (9)	0.0235 (8)	−0.0144 (8)	−0.0074 (7)	0.0014 (7)
C2	0.0295 (9)	0.0258 (9)	0.0194 (8)	−0.0125 (7)	−0.0066 (7)	0.0012 (7)
C3	0.0301 (9)	0.0301 (9)	0.0234 (9)	−0.0143 (8)	−0.0045 (7)	−0.0014 (7)
C4	0.0358 (10)	0.0354 (10)	0.0264 (9)	−0.0176 (8)	−0.0091 (8)	−0.0014 (8)
C5	0.0577 (15)	0.0605 (15)	0.0485 (13)	−0.0368 (12)	−0.0255 (11)	0.0027 (11)
C6	0.0591 (15)	0.0524 (14)	0.0437 (12)	−0.0251 (12)	−0.0296 (11)	0.0099 (10)
C7	0.0345 (10)	0.0316 (10)	0.0220 (8)	−0.0166 (8)	−0.0096 (7)	0.0051 (7)
C8	0.0395 (11)	0.0309 (10)	0.0289 (9)	−0.0104 (9)	−0.0115 (8)	0.0039 (8)
C9	0.0591 (15)	0.0308 (11)	0.0446 (12)	−0.0088 (10)	−0.0154 (11)	0.0015 (9)
C10	0.0559 (15)	0.0376 (12)	0.0446 (13)	0.0071 (11)	−0.0077 (11)	−0.0015 (10)
C11	0.0362 (12)	0.0530 (15)	0.0435 (13)	−0.0005 (11)	−0.0028 (10)	0.0044 (11)
C12	0.0337 (11)	0.0433 (12)	0.0383 (11)	−0.0108 (9)	−0.0075 (9)	0.0052 (9)
C13	0.0316 (10)	0.0327 (10)	0.0259 (9)	−0.0092 (8)	−0.0100 (8)	0.0043 (7)
C14	0.0404 (11)	0.0272 (9)	0.0255 (9)	−0.0110 (8)	−0.0103 (8)	0.0001 (7)
C15	0.0661 (15)	0.0409 (12)	0.0357 (11)	−0.0268 (11)	−0.0212 (10)	−0.0010 (9)
C16	0.0824 (19)	0.0688 (17)	0.0485 (14)	−0.0382 (15)	−0.0401 (14)	0.0050 (12)
C17	0.0601 (16)	0.0745 (18)	0.0625 (16)	−0.0296 (14)	−0.0409 (14)	0.0135 (14)
C18	0.0408 (12)	0.0444 (12)	0.0435 (12)	−0.0107 (10)	−0.0202 (10)	0.0068 (10)
C19	0.0298 (10)	0.0352 (10)	0.0259 (9)	−0.0123 (8)	−0.0075 (8)	0.0028 (8)
C20	0.0372 (10)	0.0313 (10)	0.0273 (9)	−0.0189 (8)	−0.0090 (8)	0.0001 (7)
C21	0.0471 (12)	0.0397 (11)	0.0316 (10)	−0.0215 (10)	−0.0057 (9)	0.0000 (9)
C22	0.0710 (17)	0.0501 (14)	0.0367 (12)	−0.0360 (13)	−0.0131 (11)	0.0110 (10)
C23	0.0764 (17)	0.0352 (12)	0.0554 (14)	−0.0290 (12)	−0.0331 (13)	0.0147 (10)
C24	0.0562 (14)	0.0318 (11)	0.0581 (14)	−0.0125 (10)	−0.0222 (12)	−0.0057 (10)
C25	0.0442 (12)	0.0363 (11)	0.0350 (10)	−0.0169 (9)	−0.0070 (9)	−0.0037 (9)
N1	0.0371 (9)	0.0375 (9)	0.0259 (8)	−0.0184 (7)	−0.0141 (7)	0.0032 (7)
N2	0.0430 (10)	0.0299 (8)	0.0372 (9)	−0.0191 (7)	−0.0074 (8)	0.0041 (7)
O1	0.0382 (8)	0.0393 (8)	0.0332 (7)	−0.0206 (6)	−0.0002 (6)	0.0013 (6)
O2	0.0558 (10)	0.0530 (9)	0.0247 (7)	−0.0257 (8)	0.0002 (7)	−0.0059 (6)
S1	0.0454 (4)	0.0753 (5)	0.0743 (5)	−0.0243 (3)	−0.0318 (3)	0.0007 (4)

### Geometric parameters (Å, °)

**Table:**

C1—N1	1.466 (2)	C12—C13	1.389 (3)
C1—C13	1.534 (3)	C12—H12	0.9300
C1—C7	1.560 (2)	C14—O2	1.206 (2)
C1—C2	1.592 (2)	C14—C15	1.505 (3)
C2—C14	1.531 (2)	C15—C16	1.532 (3)
C2—C19	1.547 (3)	C15—H15A	0.9700
C2—C3	1.560 (2)	C15—H15B	0.9700
C3—C20	1.516 (3)	C16—C17	1.514 (4)
C3—C4	1.523 (2)	C16—H16A	0.9700
C3—H3	0.9800	C16—H16B	0.9700
C4—N1	1.469 (2)	C17—C18	1.530 (3)
C4—C5	1.535 (3)	C17—H17A	0.9700
C4—H4	0.9800	C17—H17B	0.9700
C5—S1	1.796 (3)	C18—C19	1.537 (3)
C5—H5A	0.9700	C18—H18A	0.9700
C5—H5B	0.9700	C18—H18B	0.9700
C6—N1	1.445 (3)	C19—H19A	0.9700
C6—S1	1.830 (2)	C19—H19B	0.9700
C6—H6A	0.9700	C20—C25	1.389 (3)
C6—H6B	0.9700	C20—C21	1.394 (3)
C7—O1	1.217 (2)	C21—C22	1.378 (3)
C7—N2	1.349 (2)	C21—H21	0.9300
C8—C9	1.381 (3)	C22—C23	1.370 (4)
C8—C13	1.388 (3)	C22—H22	0.9300
C8—N2	1.400 (3)	C23—C24	1.375 (4)
C9—C10	1.382 (3)	C23—H23	0.9300
C9—H9	0.9300	C24—C25	1.385 (3)
C10—C11	1.377 (4)	C24—H24	0.9300
C10—H10	0.9300	C25—H25	0.9300
C11—C12	1.386 (3)	N2—H2	0.8600
C11—H11	0.9300		
			
N1—C1—C13	120.41 (15)	O2—C14—C2	121.75 (17)
N1—C1—C7	106.89 (14)	C15—C14—C2	118.00 (17)
C13—C1—C7	100.64 (14)	C14—C15—C16	109.25 (18)
N1—C1—C2	102.68 (14)	C14—C15—H15A	109.8
C13—C1—C2	111.37 (14)	C16—C15—H15A	109.8
C7—C1—C2	115.44 (14)	C14—C15—H15B	109.8
C14—C2—C19	108.15 (15)	C16—C15—H15B	109.8
C14—C2—C3	111.12 (14)	H15A—C15—H15B	108.3
C19—C2—C3	113.41 (15)	C17—C16—C15	113.6 (2)
C14—C2—C1	110.81 (14)	C17—C16—H16A	108.8
C19—C2—C1	113.62 (14)	C15—C16—H16A	108.8
C3—C2—C1	99.60 (13)	C17—C16—H16B	108.8
C20—C3—C4	114.20 (15)	C15—C16—H16B	108.8
C20—C3—C2	118.60 (15)	H16A—C16—H16B	107.7
C4—C3—C2	103.40 (14)	C16—C17—C18	115.7 (2)
C20—C3—H3	106.6	C16—C17—H17A	108.4
C4—C3—H3	106.6	C18—C17—H17A	108.4
C2—C3—H3	106.6	C16—C17—H17B	108.4
N1—C4—C3	104.86 (14)	C18—C17—H17B	108.4
N1—C4—C5	109.38 (17)	H17A—C17—H17B	107.4
C3—C4—C5	114.85 (16)	C17—C18—C19	114.69 (19)
N1—C4—H4	109.2	C17—C18—H18A	108.6
C3—C4—H4	109.2	C19—C18—H18A	108.6
C5—C4—H4	109.2	C17—C18—H18B	108.6
C4—C5—S1	107.24 (15)	C19—C18—H18B	108.6
C4—C5—H5A	110.3	H18A—C18—H18B	107.6
S1—C5—H5A	110.3	C18—C19—C2	116.35 (16)
C4—C5—H5B	110.3	C18—C19—H19A	108.2
S1—C5—H5B	110.3	C2—C19—H19A	108.2
H5A—C5—H5B	108.5	C18—C19—H19B	108.2
N1—C6—S1	107.08 (15)	C2—C19—H19B	108.2
N1—C6—H6A	110.3	H19A—C19—H19B	107.4
S1—C6—H6A	110.3	C25—C20—C21	117.67 (18)
N1—C6—H6B	110.3	C25—C20—C3	123.12 (17)
S1—C6—H6B	110.3	C21—C20—C3	119.15 (18)
H6A—C6—H6B	108.6	C22—C21—C20	121.1 (2)
O1—C7—N2	125.70 (17)	C22—C21—H21	119.5
O1—C7—C1	125.71 (17)	C20—C21—H21	119.5
N2—C7—C1	108.54 (16)	C23—C22—C21	120.4 (2)
C9—C8—C13	123.1 (2)	C23—C22—H22	119.8
C9—C8—N2	126.65 (19)	C21—C22—H22	119.8
C13—C8—N2	110.23 (17)	C22—C23—C24	119.5 (2)
C8—C9—C10	117.0 (2)	C22—C23—H23	120.2
C8—C9—H9	121.5	C24—C23—H23	120.2
C10—C9—H9	121.5	C23—C24—C25	120.4 (2)
C11—C10—C9	121.1 (2)	C23—C24—H24	119.8
C11—C10—H10	119.5	C25—C24—H24	119.8
C9—C10—H10	119.5	C24—C25—C20	120.8 (2)
C10—C11—C12	121.4 (2)	C24—C25—H25	119.6
C10—C11—H11	119.3	C20—C25—H25	119.6
C12—C11—H11	119.3	C6—N1—C1	117.96 (17)
C11—C12—C13	118.6 (2)	C6—N1—C4	112.11 (16)
C11—C12—H12	120.7	C1—N1—C4	111.66 (14)
C13—C12—H12	120.7	C7—N2—C8	111.73 (16)
C8—C13—C12	118.80 (19)	C7—N2—H2	124.1
C8—C13—C1	108.37 (16)	C8—N2—H2	124.1
C12—C13—C1	132.51 (18)	C5—S1—C6	88.36 (11)
O2—C14—C15	120.06 (18)		
			
N1—C1—C2—C14	154.06 (15)	C3—C2—C14—O2	−0.4 (3)
C13—C1—C2—C14	23.9 (2)	C1—C2—C14—O2	−110.2 (2)
C7—C1—C2—C14	−90.04 (18)	C19—C2—C14—C15	−50.3 (2)
N1—C1—C2—C19	−83.94 (17)	C3—C2—C14—C15	−175.42 (17)
C13—C1—C2—C19	145.89 (16)	C1—C2—C14—C15	74.8 (2)
C7—C1—C2—C19	32.0 (2)	O2—C14—C15—C16	−75.9 (3)
N1—C1—C2—C3	36.98 (16)	C2—C14—C15—C16	99.2 (2)
C13—C1—C2—C3	−93.19 (16)	C14—C15—C16—C17	−70.3 (3)
C7—C1—C2—C3	152.88 (15)	C15—C16—C17—C18	52.0 (3)
C14—C2—C3—C20	74.5 (2)	C16—C17—C18—C19	−71.4 (3)
C19—C2—C3—C20	−47.6 (2)	C17—C18—C19—C2	87.6 (2)
C1—C2—C3—C20	−168.64 (15)	C14—C2—C19—C18	−34.2 (2)
C14—C2—C3—C4	−157.92 (15)	C3—C2—C19—C18	89.6 (2)
C19—C2—C3—C4	80.01 (17)	C1—C2—C19—C18	−157.62 (16)
C1—C2—C3—C4	−41.06 (17)	C4—C3—C20—C25	−42.5 (3)
C20—C3—C4—N1	160.57 (15)	C2—C3—C20—C25	79.8 (2)
C2—C3—C4—N1	30.29 (18)	C4—C3—C20—C21	134.67 (19)
C20—C3—C4—C5	−79.4 (2)	C2—C3—C20—C21	−103.0 (2)
C2—C3—C4—C5	150.37 (17)	C25—C20—C21—C22	−2.2 (3)
N1—C4—C5—S1	22.4 (2)	C3—C20—C21—C22	−179.53 (19)
C3—C4—C5—S1	−95.20 (18)	C20—C21—C22—C23	−0.2 (4)
N1—C1—C7—O1	45.1 (2)	C21—C22—C23—C24	2.1 (4)
C13—C1—C7—O1	171.63 (18)	C22—C23—C24—C25	−1.5 (4)
C2—C1—C7—O1	−68.4 (2)	C23—C24—C25—C20	−1.0 (3)
N1—C1—C7—N2	−132.43 (16)	C21—C20—C25—C24	2.8 (3)
C13—C1—C7—N2	−5.90 (18)	C3—C20—C25—C24	−179.97 (19)
C2—C1—C7—N2	114.09 (17)	S1—C6—N1—C1	101.84 (17)
C13—C8—C9—C10	0.9 (3)	S1—C6—N1—C4	−30.0 (2)
N2—C8—C9—C10	177.8 (2)	C13—C1—N1—C6	−27.7 (2)
C8—C9—C10—C11	1.4 (4)	C7—C1—N1—C6	86.0 (2)
C9—C10—C11—C12	−1.9 (4)	C2—C1—N1—C6	−152.11 (16)
C10—C11—C12—C13	0.1 (4)	C13—C1—N1—C4	104.30 (18)
C9—C8—C13—C12	−2.7 (3)	C7—C1—N1—C4	−142.00 (16)
N2—C8—C13—C12	179.99 (17)	C2—C1—N1—C4	−20.10 (19)
C9—C8—C13—C1	171.59 (19)	C3—C4—N1—C6	128.85 (18)
N2—C8—C13—C1	−5.7 (2)	C5—C4—N1—C6	5.2 (2)
C11—C12—C13—C8	2.1 (3)	C3—C4—N1—C1	−6.0 (2)
C11—C12—C13—C1	−170.5 (2)	C5—C4—N1—C1	−129.71 (17)
N1—C1—C13—C8	123.82 (18)	O1—C7—N2—C8	−174.57 (18)
C7—C1—C13—C8	6.88 (19)	C1—C7—N2—C8	3.0 (2)
C2—C1—C13—C8	−116.00 (16)	C9—C8—N2—C7	−175.5 (2)
N1—C1—C13—C12	−63.0 (3)	C13—C8—N2—C7	1.8 (2)
C7—C1—C13—C12	−179.9 (2)	C4—C5—S1—C6	−33.23 (16)
C2—C1—C13—C12	57.2 (3)	N1—C6—S1—C5	36.70 (17)
C19—C2—C14—O2	124.6 (2)		

### Hydrogen-bond geometry (Å, °)

**Table:**

*D*—H···*A*	*D*—H	H···*A*	*D*···*A*	*D*—H···*A*
N2—H2···O1^i^	0.86	2.04	2.859 (2)	160

Symmetry codes: (i) −*x*, −*y*+1, −*z*+1.
